# Competence and Benevolence as Dimensions of Trust: Lecturers’ Trustworthiness in the Words of Italian Students

**DOI:** 10.3390/bs10090143

**Published:** 2020-09-21

**Authors:** Silvia Di Battista, Monica Pivetti, Chiara Berti

**Affiliations:** 1 Department of Psychological, Health and Territorial Sciences, The University of Chieti-Pescara, via dei Vestini 31, 66100 Chieti, Italy; chiara.berti@unich.it; 2 Department of Human and Social Sciences, The University of Bergamo, 24100 Bergamo, Italy; monica.pivetti@unibg.it

**Keywords:** authority trustworthiness, trust, competence, benevolence, higher education, qualitative methods

## Abstract

Trustworthiness includes at least two dimensions: one dimension captures the authority’s benevolence; the other captures authority’s competence. This qualitative study explores the representation of the two dimensions of authority trustworthiness: competence and benevolence. We collected free-associations about what lecturers’ competence and benevolence actually mean for Italian psychology students (*n* = 125). The data corpus was content-analyzed. Text units were categorized according to meaning using both a bottom-up strategy, with some categories stemming from the data (inductive reasoning), and a top-down strategy, with some categories following from the analysis of the relevant literature (deductive reasoning). Qualitative content analysis showed that these two dimensions overlapped. Students listed theoretically-defined competence characteristics as indications of both benevolence and competence. The same applied to benevolence. Overall, associations were grouped into two main dimensions: (1) the “can-do” dimension, describing a lecturer’s competence and social skill; (2) the “will-do” dimension, describing a lecturer’s good intentions, integrity, and personal motivation. In conclusion, the two conceptually distinct dimensions of trust are indistinguishable in the students’ words. These preliminary results are in line with scholars debating the multifactorial or mono-factorial nature of trust.

## 1. Introduction

Reviewing the literature on trust, Fink, Harms, and Mölleringin [1] defined trust as the willingness to be vulnerable in a situation of risk; and confident positive expectations among group members based on attributes of a trustee, see also [2,3]. Furthermore, trustworthiness is defined as the attributes of a trustee that inspire trust [4]. Almost all scholars share the view that trust is multidimensional regarding its components or dimensions constituting evidence of trustworthiness, e.g., [5,6]. Beyond the agreement on this definition of trust and trustworthiness, literature on this topic has been defined as being poorly integrated, widely confused and not unitary, lacking coherence, and facing a large number of challenging problems, e.g., [7]. Particularly, a predominant need remains to establish “the multi-dimensionality of trust” more concretely and contextually and solve the problems related to which dimensions of trustworthiness are distinct yet related [8]. Indeed, a complete comprehension of trust requires a depth of understanding of the components of trustworthiness. This study was intended as a preliminary exploration of the multidimensional nature of trust in the higher educational context.

### Authority Trustworthiness: Competence and Benevolence

In the review of McEvily and Tortoriello [7], authors found that the state of the art of trust measurement is rudimentary, highly fragmented, and characterized by weak evidence in support of construct validity and limited consensus on trustworthiness dimensions. They argued that it remains unclear whether or when a distinction among trustworthiness dimensions can be effectively distinguished statistically and/or meaningfully. Whipple, Griffins, and Daugherty [8] also revealed a fragmentation in the literature in the way that trustworthiness is theorized and measured. Although a remarkable amount of the relevant trust literature utilized direct and unidimensional measures (e.g., “Do you trust X?”), many researchers identified trustworthiness more specifically, recognizing its components in many different operationalizations. For instance, Lui and Ngo [9] described competence and goodwill trust as two fundamental dimensions of trustworthiness in organizations. In their study, competence included characteristics like good reputation and resources of capital and labor. Goodwill trust included characteristics like fairness in negotiations and faith. However, other researchers gave different conceptualizations of the trustworthiness components. McAllister [10] theorized a cognitive form of trustworthiness and an affective form of trustworthiness. Specifically, cognition-based trust was defined as individuals’ beliefs about others’ reliability, integrity, honesty, fairness, dependability, professional credentials, and competences. Affect-based trust reflected a referent’s responsibility, care, and concern for others’ welfare. Furthermore, the conceptualization of trustworthiness could be diversified depending on the context of study and the referent of trust. For instance, the dimensions of trustworthiness may differ if we refer to trust in peers or to trust in authorities. Indeed, Hoy & Tschannen-Moran [11] indicated that, even if every facet of trustworthiness is relevant, its effective weight could depend on the trustee, the trustor, the nature of their relationship, and the general context. 

Overall, despite this variety in theorizing and measuring components of trustworthiness, many researchers, e.g., [12,13,14,15], shared the assumption that trustworthiness should include at least two dimensions. One dimension would capture the benevolence or the “will-do” component of trustworthiness [15] also described as character-based trust [16]. The other dimension captures the competence or the “can-do” aspect of trustworthiness [15,16], also described as ability-based trust [17] and competence-based trust [12,13]. Barki and colleagues [15] tested a Boolean non-linear relationship model between the characteristics of trustworthiness and trusting behaviors. They argued that the “can-do” component describes whether the authority has the skills and abilities needed to act in an appropriate fashion, whereas the “will-do” component captures the extent to which an authority wants to help a person independent of any profit motive. In the “can-do” frame, competence has become one of the most commonly discussed components of trustworthiness; it generally captures the knowledge and skills needed to do a specific job, e.g., [12,13,16,18,19]. Sometimes, competence also refers to interpersonal competence (i.e., the skills applied in dealing with and relating to other people) [16] or to a person’s expertise and capacity to select helpful and accurate information [13]. In school settings, Chory [20] defined competence as authority expertise and knowledge in the subjects that he/she is teaching that are related to students’ sense of interactional justice. In the same context, Tschannen-Moran and Hoy [21] defined competence as the facet of trust that captured the authority skills, knowledge, and expertise needed to enhance students’ learning and well-being. Hoy and Tschannen-Moran [11] also described competence as the skills needed in situations when a person is dependent on another and that can fulfill an expectation of trust. In the “will-do” frame, benevolence was described among the most important facets of trustworthiness, particularly in the educational field [21]. Benevolence is defined as the assurance that the other will not exploit someone**’**s vulnerability or take excessive advantage of someone, even when the opportunity is available [22]. Hoy & Tschannen-Moran [11] also defined benevolence as the confidence that another party has the other’s best interests at heart and will protect them by demonstrating caring, sincerity, discreteness, fairness, goodwill, empathy, a lack of opportunism, equitability, and altruism. Chory [20] defined caring as students’ perception of authority interest in students’ needs and welfare that is related to the sense of procedural and interactional classroom justice. Thus, pertaining to educational contexts, researchers share the idea that competence and benevolence are two important antecedents of trust perceptions in authorities [21].

However, PytlikZillig, Hamm, Shockley, Herian, Neal, Kimbrough, Tomkins, and Bornstein [23] argued that relatively few empirical studies have investigated the trust-dimensionality in institutional contexts, such as university. Furthermore, little empirical work has systematically compared trust-relevant dimensions (e.g., determining the number of components of trust and which are the most similar to each other) or weighed their relations under different conditions and contexts. They found that from a measurement perspective, it may be the case that some of these conceptually-distinct components of trust are statistically or practically indistinguishable. In the university context, authors [24] manipulated and explored the effects of perceived authority’s benevolence and competence on students’ attitudes. Results showed that students from both Italy and the United States viewed a caring, competent authority as most trustworthy, and an uncaring, incompetent authority as most untrustworthy. In their quantitative study, the manipulation of competence had effects on students’ perceptions of a lecturer’s competence and smaller but significant effects on perception of the same lecturer’s good intentions. The manipulation of benevolence had effects on students’ perceptions of the lecturer’s good intentions but also a smaller effect on perceptions of his competence. This pattern of results suggested that competence and benevolence judgments are interrelated and cognitively integrated in the undergraduate students’ view. However, the quantitative studies presented above have not discussed possible differences in the qualitative content of the students’ perception of competence and benevolence. A qualitative approach could reveal something not observed. In this view, a qualitative approach has been defined as an important approach to fully understand the contents of the experience of university students [25,26]. 

Therefore, in this investigation, we aim to explore in depth the students’ representations of a faculty member’s competence and benevolence, starting from the students’ own original words and priorities. In the literature, the meaning of competence and benevolence is often unclear and overlapping. By directly asking what students had in mind when thinking of competence and benevolence, we aimed to investigate whether the overlapping present in literature could also be spotted in the representation shared by students. Do students have in mind two separate components of trustworthiness, i.e., competence and benevolence? Or do they have in mind one single dimension, with overlapping competence and benevolence characteristics? We expected students to have an overlapping mixed representation of benevolence and competence, often switching words when referring to one or to the other. We expected to find in the students’ words a mono-dimensional nature of authority trustworthiness within the university context. We also aimed to investigate the content of the subcategories of competence and benevolence as they emerged from the voices of the undergraduate students themselves. In the data analysis, we were ready to locate in the data the subcomponents present in the literature, but we were also sensitive to the priorities and dimensions spontaneously emerging from students’ associations. Do students refer to the same subcategories for competence and benevolence? Or do they have in mind specific characteristics for competence and others specific to benevolence? We expected students would confuse competence with benevolence and vice versa, and in this sense, they would use the same characteristics to describe both concepts. In this sense, we aimed to perform an exploratory qualitative study to answer our research questions concerning the overlapping between competence and benevolence.

## 2. Materials and Methods

### 2.1. Participants

Participants were 125 Italian undergraduate students enrolled on a Psychology course, aged from 20 to 42 years (Mean = 23.5; SD = 3.09), mainly female (117; 93.6%; missing for gender = 3). The short anonymous questionnaire was administered to the participants at the end of the classes. At the end of a social psychology lecture, two research assistants asked attending students to voluntarily fill in the questionnaire, explaining the purpose of the study, naming the researchers involved, and describing the potential use of the collected data. No remuneration was offered. Respondents were ensured anonymity. The lecturer left the room during data collection. Those students unwilling to take the questionnaire simply left the room. Purposive sampling is commonly used in qualitative research [27], and it generally indicates that participants are selected on the basis of their knowledge and verbal eloquence to describe the subculture or the issue being investigated [28]. The questionnaire took approximately 15 min to fill in. The research was compliant with the Code of Ethics of the Italian Psychology Association [29] that draws inspiration from the Declaration of Helsinki. No Institutional Review Board for Psychology research was available at the affiliations of the social psychology researchers involved in the study (i.e., University of Chieti-Pescara), so no request for approval was submitted. 

### 2.2. Materials

Participants filled in a written questionnaire including a free-association task, a data collection method commonly used in social sciences, e.g., [30,31]. Participants were instructed to write the first five words or short sentences coming into their minds when prompted by the stimulus word: “a competent lecturer”, “a benevolent (well-intentioned) lecturer”, “an incompetent lecturer”, and “a malevolent (or ill-intentioned) lecturer”. Giving a stimulus word and asking the respondent to freely associate what ideas came into his or her mind gives relatively unrestricted access to mental representations. The stimulus words used in the questionnaire were inspired by the relevant psychosocial literature on trustworthiness e.g., [3,4,11]. 

The last section of the questionnaire included socio-demographic questions concerning age, gender, university proficiency, and ethnic background. 

### 2.3. Analysis of the Material

After the first round of data collection, we inspected the word associations produced by participants, and we considered that we had already reached the saturation for this population, as no new concepts/images emerged from the data. Hence, we did not perform a second round of data collection.

The data corpus was composed of four sets of word associations or dictionaries, one for each target definition: “a competent lecturer”, “a benevolent (well-intentioned) lecturer”, “an incompetent lecturer”, and “a malevolent (or ill-intentioned) lecturer” (In Italian, nouns and adjectives have gender, for instance: “il professore” for male lecturers, “la professoressa” for female lecturers. To obtain a more homogeneous dictionary, we decided not to consider the masculine and feminine gender of nouns and adjectives in the analysis and to transform any words from the feminine to the masculine gender.). Verbal material was transcribed verbatim. Table 1 describes the total number of words/sentences per dictionary and the average number of word/sentences per participant, per dictionary.

The words produced were first processed to make the corpus of words more uniform and less ambiguous. For instance, terms that differed only in grammatical form (gender and singular/plural) were grouped together. The meaning of some words in the context of faculty members’ behavior or attitude was not understandable, and therefore they were deleted from the data corpus (e.g., “frustration”, “young”) The data corpus was content-analyzed according to procedures outlined by Dey [32] and Flick [33]. The participants’ single productions, being a single word or a sentence or a set of statements, were considered a text unit. Categorization of the text units into themes was conducted using both a bottom-up strategy, with some categories stemming from the data, after a repeated reading of the interview material (inductive reasoning), and a top-down strategy, with some categories following from the analysis of the relevant literature (deductive reasoning). The same text units could be grouped into two or more categories if conveying two or more different meanings/characteristics regarding the target. As part of a training phase, two judges coded the verbal production independently and produced their own coding schemes. One judge was an expert in trustworthiness and the other was naïve on the issue, to assure that different point of views could emerge during the coding process. Subsequently, the two judges met and compared their coding schemes, discussing their rationale in classifying particular text units within specific themes as well as the appropriateness of the theme labels. Inconsistencies and disagreements between the two coders were resolved through discussion with a third judge, experienced in qualitative content analysis. In this phase, the three judges generated the final coding scheme, including categories and subcategories. The three judges agreed on the content of each category/subcategory and shared the same definitions of category labels. New categories were generated through discussions with the third judge. For instance, after careful examination of the many text units originally coded by the two judges into the category “other”, the three judges agreed to create a new category named “management of the classroom environment”. Next, each of the two original judges coded the entire material independently in a systematic fashion, according to the shared common coding scheme. Finally, inconsistencies and disagreements between the two judges were solved through discussion with the experienced third judge [34].

## 3. Results

When students evaluate a lecturer in terms of his/her competence, they take into consideration their previous experiences with both competent and incompetent lecturers. For this reason, we decided to analyze together the data corpus produced via the stimulus word “competent lecturer” and via the stimulus word “incompetent lecturer.” This way, we hoped to have a more comprehensive picture of the students’ view of the lecturer’s competence, both in the negative and positive side. The same reasoning applies when judging the lecturer’s benevolence. We analyzed together the dictionaries relating to “benevolent” and “malevolent” lecturer. The analyses were run separately for competent/incompetent lecturer, on the one hand, and for benevolent/malevolent lecturer on the other hand. For clarity, results are presented divided into the representation of the “competent/incompetent lecturer” and the representation of the “benevolent/malevolent lecturer”.

### 3.1. Representation of the Lecturer: the “Can-Do” and the “Will-Do” Dimensions

Two main categories emerged when categorizing the text units relating both to a competent/incompetent lecturer and the text units relating to a benevolent/malevolent lecturer: “can-do” and “will-do” [15]. The “can-do” described a lecturer as characterized by (1) “hard competencies” relating to the knowledge of the subject he/she was teaching; the lecturer was described as competent in terms of expertise and knowledge, the ability to explain the main topics of his/her course, of being clear, well-prepared, intelligent, and acculturated; (2) the management of classroom environment; the lecturer was described in terms of interpersonal skills relating to the involvement of students during lessons. The “will-do” grouped three aspects relating to the lecturer’s good intentions, care and concern, and personal motivation. Specifically, the “will-do” dimension consisted of: (3) benevolence; the lecturer was someone with a positive attitude, available, able to be empathetic and sociable with students, and someone with strong social skills; (4) morality; the lecturer was represented in terms of professional integrity, that is equity, reliability, and loyalty; (5) motivation; the lecturer was described as being passionate in teaching, motivated at work, and able to transmit his/her enthusiasm to students. 

**Figure 1:**
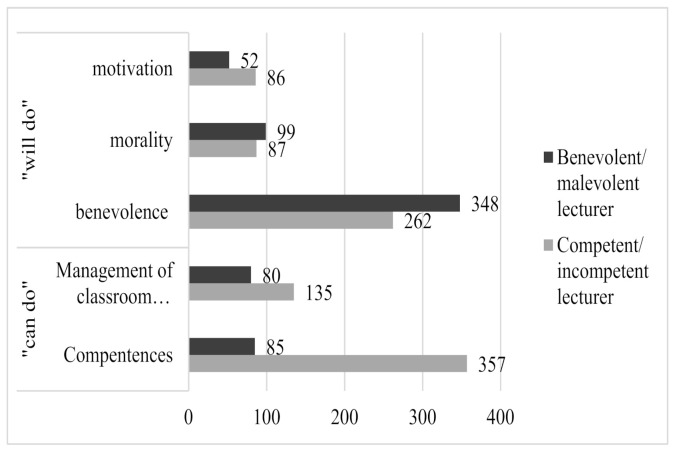
Distribution of the text units elicited by the competent/incompetent and benevolent/malevolent lecturer, grouped into the relevant categories. Note. *n* = 125.

Whereas the same five categories emerged in the two dictionaries, that is competent/incompetent lecturer and benevolent/malevolent lecturer, the number of text units grouped into each category varied across dictionaries. Figure 1 provides a graphic representation of the main themes pertaining to the competent/incompetent lecturer and to the benevolent/malevolent lecturer, as well as the number of text units grouped into each category and subcategory. On one hand, when asked about the competent/incompetent lecturer, participants focused mainly on the “can-do” dimensions, which are competences and management of the classroom environment. As for the competent/incompetent lecturer, the most frequent categories were competence (no. of text units = 357), belonging to the “can-do” dimension, followed by the benevolence category (no. of text units = 262), belonging to the “will-do” dimension. For instance, when asked about a competent lecturer, participant ^#^120 reported: “(he/she) is available for further eventual clarifications and discussion”, later coded as “benevolence”. When asked about an incompetent lecturer, participant ^#^123 reported that: “(he/she) is apparently a layabout”, later coded as “personal motivation.” 

On the other hand, when asked about benevolent/malevolent lecturer, participants focused more on the “will-do” dimension”, that is benevolence, morality, and motivation. As for the benevolent/malevolent lecturer, the most frequent category was benevolence (no. of text units = 348), followed by morality (n. of text units = 99), both pertaining to the “will-do” dimension. Remarkable was the number of text units elicited by the benevolent/malevolent prompt word and then grouped into the “can-do” dimensions: competence (n. of text units = 85) and management of classroom environment (n. of text units = 80). For instance, when asked about a benevolent lecturer, participant ^#^5 reported: “to have a great study material”, later coded as “competence” and “to share his/her material”, later coded as “competence”. When asked about a malevolent lecturer, participant ^#^44 reported: “unclear (in explaining lectures)”, later coded as “competence.”

In sum, the students’ view of competent/incompetent behaviors of their hypothetical university authority was confused, with students producing words/sentences belonging both to the “can-do” dimension and to the “will-do” dimension for the same stimulus word. The same applies to the representation of the benevolent/malevolent authority. Students did not differentiate between competence and benevolence when describing their university authorities.

In the next section, we describe in depth the content of the representation of the “competent/incompetent lecturer” and then the content of the representation of the “benevolent/malevolent lecturer”.

### 3.2. The Competent/Incompetent Lecturer

#### 3.2.1. The “Can-Do” Dimension

A lecturer’s competence was discussed in terms of expertise and knowledge, ability to explain the main topics of his/her course, and being clear, prepared, intelligent, and up to date. A competent lecturer had teaching experience and was pragmatic. On the contrary, an incompetent lecturer was unprepared and superficial in classes, not updated. He/she was unable to explain the subjects during lectures and did not parallel the textbooks and other lecture materials in class. As expected, this theme relating to teaching skills contained the majority of associations produced by participants when asked to describe a competent/incompetent lecturer. Examples of words/sentences for each theme and subtheme were presented in Table 2.

However, a competent lecturer was also surprisingly described as someone who had leadership skills to manage the classroom environment. He/she was charismatic, authoritative, a go-getter, brilliant, fascinating, and charming. Participants described competence in terms of interpersonal skills relating to the involvement of students during lessons. On the contrary, students complained about an incompetent lecturer who was described as boring, distracted, rigid, insecure, and passive. Students criticized the implementation of direct lectures, with the lecturer describing the content of the slides in class and not involving the audience in any form of interaction.

#### 3.2.2. The “Will-Do” Dimension

A lecturer’s competence was also surprisingly represented in terms of good intentions, concern, sociability, and personal motivation. Specifically, the “will-do” dimension consisted of (1) benevolence, (2) morality, and (3) personal motivation. As for benevolence, a competent lecturer was someone with a positive attitude, able to be empathetic and sociable with students. This dimension related to the social skills of faculty members. He/she was kind, respectful, and approachable. An incompetent lecturer was described as unlikable, cruel, with bad intentions and motives, and unsociable. He/she was cold, superficial, not available, and hateful. As for morality, a competent lecturer was represented in terms of professional integrity, that is, equity, reliability, and loyalty. He/she was respectful of others’ points of view, fair, objective, and not discriminatory. An incompetent lecturer was described as being impolite, unfair, and inconsistent. He/she was often late for lessons, was not doing his/her job properly, and had pet students. As for personal motivation, a competent lecturer was described as being passionate at teaching, motivated at work, and able to transmit his/her enthusiasm to students. He/she loved his/her job, loved teaching, and was fond of his/her subject. An incompetent lecturer was described as not motivated at work or not passionate at teaching. He/she was lazy, dissatisfied, lethargic, not passionate, and uninterested in what he/she was doing.

### 3.3. The Benevolent/Malevolent Lecturer

#### 3.3.1. The “Will-Do” Dimension

A benevolent lecturer was described as someone with a positive attitude, able to be empathetic and sociable with students. This dimension was related to the social skills of faculty members. He/she was approachable, altruistic, empathetic, nice, indulgent, and calm. On the contrary, a malevolent lecturer was not nice, bad, unfriendly, and aggressive. He/she was not listening to students’ requests, considered the students as inferior persons, and was putting students in difficulty on purpose. Table 3 provides some examples of words/sentences for each theme and subtheme. As for morality, the lecturer was respectful, punctual, fair, polite, and neutral. On the contrary, a malevolent lecturer was someone who was unjust and inconsistent. He/she was unfair, had preferences and was biased, not professional, vindictive, and unkind. As for personal motivations, the lecturer was described as being passionate in teaching, motivated at work, and able to transmit his/her enthusiasm to students. He/she loved his/her job, was trying to do his/her best, and was keen. On the contrary, a malevolent lecturer had no motivation at work, he/she was bored and lethargic, and had no passion for what he/she was doing.

#### 3.3.2. The “Can-Do” Dimension

As for competence in terms of expertise and knowledge, the benevolent lecturer was also surprisingly described as being clear, prepared, intelligent, and up to date. He/she provided good examples during classes, was experienced and pragmatic. He/she gave clear indications about examinations and theses and was well-organized. On the contrary, a malevolent lecturer was someone who did not follow the teaching material, was unprepared, and was unable to explain the lesson. He/she was hasty, rude, pushover, not updated, inaccurate, and unclear. As for management of classroom environment, a benevolent lecturer was someone with good interpersonal skills and enticing. He/she was curious, captivating, resolute, and purposeful. He/she was able to involve students in the lessons and delivered light and entertaining lessons. On the contrary, a malevolent lecturer was described as cold, authoritative, boring, heavy, rigid, not creative, and inflexible (see Table 3). The content of this category for the benevolent/malevolent prompt word was similar to the content of the same category for the competent/incompetent prompt word. 

## 4. Discussion

In this qualitative investigation, we have explored the Italian students’ representation of two frequently described facets of trustworthiness in education contexts, namely competence and benevolence, and their possible overlap. Relatively few empirical studies have explored the trust-dimensionality in institutional contexts, such as university [23], and few empirical investigations, e.g., [24], have suggested the extent to which competence and benevolence judgments are interrelated and integrated. We collected qualitative data about what authorities’ competence and benevolence mean in the students’ words. As far as we know, no empirical studies have applied such a “bottom-up” approach based on qualitative data from naïve participants to study authority trustworthiness. The qualitative content analysis of Italian students’ descriptions of faculty behavior confirmed the extent to which trust components were interrelated: students listed theoretically-defined competence/incompetence characteristics as indications of both benevolence/malevolence and competence/incompetence, and theoretically-defined benevolence/malevolence characteristics as indications of both competence/incompetence and benevolence/malevolence. In line with research that has found limited consensus on trustworthiness dimensions and uncertainty related to which dimensions of trustworthiness are distinct yet related, e.g., [7,8], we found that the two dimensions of trustworthiness indeed overlapped in the students’ words. Furthermore, some aspects of trustworthiness emerged that were not strictly related to definitions of competence and benevolence. For instance, a category describing whether or not a lecturer had or did not have the leadership skills to manage the classroom environment also emerged. In this case, a competent/incompetent lecturer was described as being charismatic, authoritative, a go-getter, brilliant, fascinating, and charming vs. boring, distracted, rigid, insecure, and passive, whereas a benevolent/malevolent lecturer was described as someone with good interpersonal skills, enticing, and able to involve students in the lessons vs. someone who is cold, authoritative, boring, heavy, rigid, not creative, and inflexible. This aspect related to the management skills can be theoretically located in the “can-do” dimension of trustworthiness [15]. These descriptions mirror Gabarro’s definition [16] of interpersonal competence that described people’s abilities and skills at building social relationships that help interaction and transactions. Sometimes students also referred to the “morality” or “integrity” of a lecturer’s behavior, defined as the desire to be consistent with a set of ethics or rules. In this case, a competent/incompetent lecturer was described as being respectful of others’ points of view and professional vs. impolite, absent, or unfair, whereas a benevolent/malevolent lecturer was described as someone who is respectful, polite, and fair vs. someone who has preferences and is biased. In the literature, the concept of morality or integrity is frequently described as one important dimension of human perception, [35,36] and it is sometimes described as one aspect of the “will-do” component of trustworthiness [3,4,15]. Some scholars proposed that benevolence is strictly distinct from integrity, but other evidence suggests these two aspects of trustworthiness are highly related especially for the relatively short-term authority relationships typical of the university context [23]. Finally, a category describing a lecturer’s motivation to work hard and to transmit his/her enthusiasm to students was found. In this case, a competent/incompetent lecturer was described as someone who loves his/her job and teaching vs. someone who is apathetic and dissatisfied, whereas a benevolent/malevolent lecturer was described someone who is passionate vs. someone who is lethargic and bored. Other researchers found that one’s degree of passion for and attachment to his/her expertise facilitates knowledge acquisition and transfer [37].

All these aspects were categorized into two dimensions of lecturer trustworthiness, namely “can-do” and “will-do” [15]. In the literature, Barki and colleagues [15] referred to these two key motivational determinants of trust as the “can-do” component of trustworthiness, whether the trustee has the skills and abilities needed to act appropriately and the ‘‘will-do’’ component, whether the trustee will choose to use those skills and abilities to act in the best interests of the trust giver. This is also in line with the two dimensions of organizational trust of Whipple and Frankel [38]—based on Gabarro’s [16] intra-organizational work—namely: (1) competence-based trust (i.e., specific competence and interpersonal competence) trust; (2) character-based trust (i.e., integrity, identification of motivations, consistency of behavior, and openness). In our results, these two main categories emerged when categorizing the text units related both to a competent/incompetent lecturer and the text units related to a benevolent/malevolent lecturer. In both of them, it was possible to identify competence/incompetence issues relating to the knowledge of the subject he/she was teaching and benevolence/malevolence issues. 

In sum, the students’ representations of competent/incompetent behaviors of their hypothetical university authority were mixed, with students producing words/sentences belonging to the “can-do” dimension and to the “will-do” dimension for the same stimulus word. The same applied to the representation of the benevolent/malevolent authority. Students did not differentiate well between competence and benevolence when describing their university authorities. However, they gave an articulated and comprehensive representation of their university authorities, adding some aspects such as their motivation to teach and capacity to arouse the students’ curiosity. University students considered that a trustworthy university authority should be characterized by both the knowledge of subject he/she was teaching and by the lecturer’s good intentions; they appreciated aspects of good class management, morality, and motivation to teach. In line with PytlikZillig and colleagues [23], who suggested that it may be the case that the conceptually-distinct facets of trust are statistically or practically indistinguishable, we found that competence and benevolence overlap in the students’ words. We confirm the literature’s assumptions that confusion endures about the meaning of trust and the independence of its facets [7].

The overlap between trust-relevant concepts likely depends on the specific contexts examined. For instance, Schoorman, Mayer, and Davis [39] noted that, whereas judgments of competence could form relatively quickly in the course of a relationship, benevolence judgments needed longer to develop. They argue that, in samples where the parties had longer-lasting relationships, multiple components of trustworthiness were more likely to be separable factors. In the Italian university context, student–lecturer relationships are short-lived, formal, and limited to a few areas of class time, exams, and student tutorials. Furthermore, our results suggest that, in an educational context, both dimensions are relevant for student–lecturer relationships. In other words, for many students, a lecturer who behaves benevolently might be, by definition, competent; and the opposite. 

Overall, in educational contexts, both dimensions of competence and benevolence contribute to students’ assessment of the educational authorities’ trustworthiness [24] and are vital for number of positive outcomes, e.g., [11,21,40]. Trustworthiness is fundamental in regard to the processes required for the healthy functioning of schools and academies, and it predicts students’ engagement [21,24,25,26,27,28,29,30,31,32,33,34,35,36,37,38,39,40,41]. For instance, Mitchell and colleagues [42] argued that if students believe that they can trust their teachers, they are more actively engaged with instructional goals and more likely to cooperate for cultivating safe schools. Analyzing qualitative data collected at a Finnish university, Kosonen and Ikonen [43] demonstrated that, by showing trustworthiness, leaders promoted the followers’ organizational engagement. However, students’ perception about lecturers as legitimate figures of authority have been challenged in the last years [44]. Our study results could make clearer what students mean by a trustworthy authority in order to know how to manage educational context and encourage students as to the legitimate use of authority rather than the arbitrary use of power. 

One novelty of the study lies in the discovery of the importance of faculty members’ social skills when judging trustworthiness. Being able to manage a university class, involve students during a lecture, and transmit passion for the subject calls for a deep reflection on the faculty members’ educational approach to classes. It also calls for paying more attention to candidates’ social skills when recruiting and evaluating lecturers in the Italian university system. 

As for the research strategy, this is a qualitative study by nature, but the data analysis strategy somehow falls in between the qualitative and quantitative approach, as the data were qualitatively content-analyzed by coding each text units into categories according to meaning. We then counted down the number of text units coded into each category, in view of the fact that the categories named most often are somehow more relevant for the participants. By linking qualitative and quantitative results, we consider that we have enriched our understanding of the issue under investigation [45]. We have studied both the meaning of competence and benevolence in the sampled population and have also observed the distribution of the different subcategories for the benevolence and competence individually. This way, we were also able to compare the relative distribution of the text units for each subcategory across the two data corpora: the one emerging from the prompt-word “competence” and the one from the prompt-word “benevolence.” This data analysis strategy linking both qualitative and quantitative results is quite common in social psychology research [46,47,48].

The study had some important limitations that ought to be kept in mind when interpreting the results. One limit is the sample composition, made up mainly of female students from central and southern Italy, and in the convenient nature of the sample, leading to low representativeness of the sample. Future research should explore the same topic in a more representative sample of students. It is necessary to emphasize that this is a preliminary study that should be extended to a larger population. However, this study adds evidencs and offers a new approach to observing the meaning of trust. Furthermore, we did not counterbalance the presentation of the stimulus words in the free-association task. Invariably, all the participants were given “competent lecturer” as the stimulus word first of all, and “benevolent (well-meaning) lecturer” afterwards. Future research should investigate the possible order effects that are of special concern in within-subject designs. Finally, future studies should take into account the role of the lecturer’s gender in the perception of trust. In Italian, nouns (such as lecturer) have gender, for instance: “il professore” for a male teacher, “la professoressa” for a female teacher. The instrument was correctly administered asking for a “competent/incompetent” or “benevolent/malevolent”—male or female—lecturer. However, in order to obtain a more homogeneous dictionary, we decided not to consider the masculine and feminine gender of nouns in the analysis. Following some notions from the stereotype content model SCM [49], future studies could explore the perception of trust in the case of male and female lecturers. 

## 5. Conclusions

This study provided a qualitative investigation of Italian students’ representation of lecturers’ competence and benevolence, and their possible overlap. Results have shown that the students’ representations of lecturers’ competent/incompetent behaviors were mixed, with students producing words/sentences belonging to the “can-do” dimension and to the “will-do” dimension for the same stimulus word. The same applied to the representation of the lecturers’ benevolent/malevolent behaviors. In sum, students did not differentiate well between competence and benevolence when describing their university authorities. However, they gave an articulated representation of their university authorities, adding some aspects such as lecturers’ motivation to teach and capacity to arouse the students’ curiosity, leadership skills, and morality concerns. This study offers a new lens through which to study the meaning of authority trustworthiness from the students’ point of view. 

## Figures and Tables

**Table 1:** Number of words/sentences produced by participants for each target definition.

HeadingTarget	Competent	Benevolent	Incompetent	Malevolent
Number. of words/sentences	525	367	414	315
Number of words/sentences per participant	4.2	2.9	3.4	2.5

Note. *n* = 125.

**Table 2:** Examples of words/sentences for competent/incompetent lecturer, by the “can-do” and the “will-do” categories.

Heading Categories	“Can-Do”		“Will-Do”		
	Competences	Management of Classroom Environment	Benevolence	Morality	Personal Motivation
Competent lecturer	Up to date	Capable of keeping students’ attention high	Nice	Not discriminatory	Fond of his/her subject
	Clear	Able to capture students’ attention	Willing to provide explanations	Respectful of others’ points of view	He/she loves his/her job
	Prepared	Charismatic	Approachable	Professional	He/she loves teaching
Incompetent lecturer	Not updated	Incapable of social interaction	Not available	Impolite	Feels like doing nothing
	Not prepared	Lacking initiative	Pretentious	Absent	Apathetic
	Not well-read	Dull	Quick-tempered	Unfair	Dissatisfied

**Table 3:** Examples of words/sentences for benevolent/malevolent lecturer, by the “can-do” and the “will-do” categories.

Heading Categories	“Can-Do”		“Will-Do”		
	Competences	Management of Classroom Environment	Benevolence	Morality	Personal Motivation
Benevolent lecturer	Intelligent	Curious	Approachable	Punctual	He/she loves his/her job
	He/she has experience	Captivating	Altruistic	Respectful	Passionate
	Pragmatic	Resolute	Empathetic	Polite	Passionate about explaining
Malevolent lecturer	Unclear	Authoritative	Egoistic	Unfair	Lethargic
	Unprepared	Heavy	Bad	He/she has preferences	Bored
	Superficial	Dull	Critical	He/she is biased	He/she has no passion for what he/she is doing
